# Silk Fibroin Seed Coatings: Towards Sustainable Seed Protection and Enhanced Growth

**DOI:** 10.3390/polym16233281

**Published:** 2024-11-25

**Authors:** Feng Jin, Zhengrong Guan, Jiahao Zhang, Zhigang Qu, Shengjie Ling, Leitao Cao, Jing Ren, Ruoxuan Peng

**Affiliations:** 1 Shengzhou Mulsun Biotech Co., Ltd., 99 Jincan Road, Shengzhou 312499, China; jeffjin@mulsun.com (F.J.); quzhigang2019@163.com (Z.Q.); 2 School of Physical Science and Technology, ShanghaiTech University, 393 Middle Huaxia Road, Shanghai 201210, China; jacky_070218@outlook.com (Z.G.); zhangjh6@shanghaitech.edu.cn (J.Z.); caolt@shanghaitech.edu.cn (L.C.); renjing@shanghaitech.edu.cn (J.R.); 3 State Key Laboratory of Molecular Engineering of Polymers, Department of Macromolecular Science, Fudan University, Shanghai 200433, China; 4 State Key Laboratory of Advanced Medical Materials and Devices, ShanghaiTech University, Shanghai 201210, China; 5 Shanghai Clinical Research and Trial Center, Shanghai 201210, China

**Keywords:** seed coating, silk fibroin, antimicrobial peptides, eco-friendly

## Abstract

Seed coating technology is vital in agriculture, enhancing seed protection and growth. However, conventional coatings often include chemical fungicides that pose environmental risks, highlighting the need for sustainable alternatives. This study explores silk fibroin (SF), a natural biopolymer with excellent film-forming properties, as a potential seed coating agent, addressing its antimicrobial limitations by combining it with the commercial agent CRUISER^®^ and the antimicrobial peptide Nisin. Experimental methods included solution stability analysis, Fourier-transform infrared spectroscopy (FTIR), scanning electron microscope (SEM), and growth assessments of wheat seeds. Findings reveal that silk fibroin-CRUISER^®^ (SC) composites form stable β-sheet structures, enhancing the coating’s mechanical strength. SF-based coatings improved seedling emergence rates (up to 1.65-fold), plant height (up to 1.05-fold), and root growth (up to 1.2-fold), especially under cold stress. The addition of Nisin further significantly boosted the antibacterial properties, providing sustained pathogen inhibition (*p* < 0.01). Identifying the optimal concentration of SF was essential for achieving a balance between protection and breathability, a key factor for industrial application. This research provides valuable insights into the development of eco-friendly seed coatings, presenting a viable and sustainable alternative to traditional chemical-based options in agricultural practices.

## 1. Introduction

Seed coating technology is revolutionizing agricultural production by strategically applying advanced functional materials to the seed surface. This innovative approach not only shields seeds from detrimental environmental factors but also catalyzes their germination and growth, thereby enhancing crop performance and resilience [1,2,3]. The coating prevents mechanical damage, oxidation, and ultraviolet radiation, thereby enhancing seed survival and germination rates [4,5]. Additionally, the coating materials often contain nutrients and moisture, which provide essential support during the germination and growth phases, ensuring successful sprouting under suitable conditions [6,7].

Traditional seed coating materials typically include polymers, nutrients, and biopesticides [8]. For instance, polymer materials such as polyvinyl alcohol and polyacrylic acid possess excellent degradability and water retention properties, effectively safeguarding seeds from environmental stressors [9,10]. Furthermore, the coating materials often incorporate organic fertilizers and trace elements to support healthy growth during the early stages [11]. To enhance the seeds’ resistance to pests and diseases, insecticides and fungicides are also commonly added to the coating formulations [12,13].

Despite the significant role of seed coating technology in agriculture, several challenges remain. Some commonly used seed coating materials, such as fungicides and non-degradable polymer additives, may pose environmental risks, potentially impacting soil quality with prolonged use [5,14,15,16]. Moreover, excessively thick or hard coatings can hinder normal seed germination and reduce crop yield [15]. The composition and quality of the coating materials are also critical factors affecting the seeds’ resistance to diseases and pests and their adaptability to environmental conditions [17].

In recent years, silk fibroin (SF) has emerged as a promising material for seed coating research due to its excellent film-forming properties and biocompatibility [18,19,20,21]. SF can form stable films that protect seeds from oxidation, humidity fluctuations, and ultraviolet exposure [22,23,24]. Additionally, SF films can incorporate nutrients to further promote seed germination and growth [25]. However, current applications of SF in seed coatings primarily focus on its use as a single component, with limited research on its combination with other seed coating agents.

The integration of SF with other seed-coating components represents an essential trend in the industry. This is because effective seed-coating agents typically consist of intricate formulations that incorporate various functional additives. These additives serve to fulfill a range of objectives, including seed protection and enhancement of growth [1,2,3]. At the same time, SF alone might not possess such multifunction, such as adequate antibacterial capabilities [26]. Additionally, SF aqueous solution is prone to denaturation under the influence of alcohol, salt, and physical stress, leading to the secondary structure of the silk protein molecular chain from a random coil (easily soluble in water) to a β-sheet structure (serrated and arranged in layers, insoluble in water) [27]. This alteration in conformation could potentially impact the preparation, storage, and application of SF-based seed-coating agents. Therefore, exploring suitable functional components and ensuring the compatibility between SF and functional components has become a critical issue in developing powerful seed coating solutions [28,29].

To address this, the present study first explored the feasibility of combining SF with CRUISER^®^, a commercial seed coating agent, and validated its effectiveness on key metrics such as emergence rate, plant growth, and drought resistance. However, the fungicide components in CRUISER^®^ present potential environmental risks. Therefore, this study introduced an antimicrobial peptide as an alternative to toxic fungicides, such as Nisin, a natural, safe, and non-toxic antimicrobial peptide produced by *Lactococcus lactis* [30], to verify its antibacterial efficacy. This approach offers a new direction for the design of eco-friendly seed coating agents.

## 2. Materials and Methods

### 2.1. Materials

Anhydrous calcium chloride (CaCl_2_, AR) and sodium bicarbonate (NaHCO_3_, AR) were obtained from Meilunbio Co., Ltd., Dalian, China. Ethanol was obtained from Sinopharm Chemical Reagent Co., Ltd., Shanghai, China. CRUISER^®^ was obtained from Syngenta Group Co., Ltd., Shanghai, China. Rhodamine B was obtained from Shanghai Aladdin Biochemical Technology Co., Ltd., Shanghai, China. *Bombyx mori* (*B. mori*) silkworm cocoons were provided by the Shengzhou Musang High-tech Co., Ltd., Shengzhou, China. Wheat seeds were obtained from Hebei Letu Seed Industry Co., Ltd., Shijiazhuang, China. Nisin was obtained from Shandong Yuantai Biological Engineering Co., Ltd., Rizhao, China.

### 2.2. Preparation of the SF Solution

A total of 20 g of silkworm cocoons was boiled twice for 30 min in an aqueous solution of 0.5 wt% NaHCO_3_. The degummed silk fibers were washed in deionized water and then placed in an oven at 60 °C for 12 h for drying. The three-component solution of CaCl_2_, ethanol, and H_2_O (Deionized Water) was configured according to the molar ratio of 1:2:8, with a total weight of 180 g. A total of 20 g of degummed silk fibers were added into a ternary solvent and heated in a water bath at 60 °C for 4 h to completely dissolve and obtain an SF solution. The impurities were removed by filtration with three layers of gauze. After the temperature of the SF solution dropped to room temperature, the solution was put into several dialysis bags for dialysis. The water was changed every 6–8 h for 3 days, and the solution of SF with a concentration of about 5% was obtained.

### 2.3. Preparation of Seed Coating Agent

Different proportions of seed coating agents were prepared using CRUISER^®^ solution and SF solution (Table 1). After adding each solution in proportion, mix directly and stir evenly. Each 20 mL of coating agent can coat 1 kg of wheat seeds.

### 2.4. Preparation of SC Composite Film

Following the casting of the seed coating agent onto a polystyrene mold (40 × 40 mm), it was then placed in a fume hood to allow for solvent evaporation about 24 h to form a uniform film.

### 2.5. Preparation of Seeds Coating with SC Composite System

Put the coating agent into the coating bag (ziplock bag) and make the coating agent evenly distributed in the bag. Pour the seeds into a coating bag and inflate the bag. Shake the bag in an unfixed direction to evenly cover the seeds. Tip the coated seeds out to dry [31].

### 2.6. The Sowing of Coated Wheat Seeds and the Record of the Growth

Seven different seed treatments for wheat seeds: SF-1, SF-2, SF-3, CRUISER^®^, SC-1, SC-2, and SC-3. Untreated wheat seeds served as the control group.

The seeds were planted in seed trays (5.5 cm length × 5.5 cm width × 5 cm height) and filled halfway with a 1:1 mix of desalinated coir brick and vermiculite. There were 18 trays, and, in each tray, 10 wheat seeds were sown, which were then covered with vermiculite. The trays were watered from the bottom, and the seedlings were grown under a 12 h light/12 h dark cycle at 22 °C during the day and 18 °C at night.

Average emergence rates: the number of wheat seedlings in 18 trays was counted, and the ratio of the number of seedlings to the total number of wheat seeds (total 180) was taken as the average emergence rates. Recorded the average emergence rates on the 7th and 14th days after seeding.

Plant growth: On the 14th and 21st days after sowing, according to plant height, leaf area, and leaf length, the total plant growth in each group was evaluated visually. The growth of the control group was 100, and the growth of the other seven groups was scored after comparison with the control group.

Average plant height: The average plant height (cm) in each tray was measured on the 14th day. Each group was measured for 10 trays.

Root growth: On the 21st day after sowing, according to the root length and distribution, the root growth in each tray of each group was evaluated visually, photographed, and recorded. The growth of the control group was 100, and the growth of the other seven groups was scored after comparison with the control group.

### 2.7. The Sowing of Coated Wheat Seeds Under Cold Stress Conditions and the Record of the Growth

Coated wheat seeds treated with SF-1, SF-2, SF-3, CRUISER^®^, SC-1, SC-2, and SC-3. Uncoated wheat seeds were used as control. It is worth noting that the seeds were planted outdoors under cold stress (10 °C /4 °C in the day/night, 12 h light/12 h dark) [32,33]. The number of wheat seedlings in 18 trays was counted, and the ratio of the number of seedlings to the total number of wheat seeds (total 180) was taken as the average emergence rates. Recorded the average emergence rates on the 5th day after seeding.

### 2.8. The Sowing of Coating Wheat Seeds Under Delayed Sowing Conditions and the Record of the Growth

Coated wheat seeds treated with SF-1, SF-2, SF-3, CRUISER^®^, SC-1, SC-2, and SC-3. Uncoated wheat seeds were used as a control. It is worth noting that the sowing time was 18 days and 37 days after the coating production. Other culture conditions were consistent with normal culture. The number of wheat seedlings in 18 trays was counted, and the ratio of the number of seedlings to the total number of wheat seeds (total 180) was taken as the average emergence rates. Recorded the average emergence rates on the fifth and seventh days after seeding.

### 2.9. Preparation of SF/Antimicrobial Peptide Composite System

The 4% Nisin solution was obtained by dissolving Nisin in H2O. The 5% SF-4% Nisin solution was obtained by dissolving Nisin in 5% SF solution.

### 2.10. Detection of Antibacterial Effect of SF/Antimicrobial Peptide Composite System

Use solid agar plate diffusion to test antimicrobial activity [34]. Firstly, the indicator strains Staphylococcus aureus and Bacillus subtilis, cultured to the logarithmic growth phase, were mixed with melted LB agar medium (a starting OD_600_ of 0.05). We put pieces of paper disks on the agar plate, and each coating agent was added to the paper disk, such as SF, Nisin, SF/Nisin, and Kana. The plates were cultured at 37 °C for 20 h. We observed and measured the diameter of the zone of inhibition (DZI, in mm) to determine the antibacterial effect of the SF/Nisin composite system.

### 2.11. Characterization

The microstructure of the sample was obtained using a scanning electron microscope (SEM) (JSM-7800, JEOL Ltd., Tokyo, Japan) available at the Center for High-Resolution Electron Microscopy, SPST, ShanghaiTech University.

Fourier-transform infrared spectroscopy (FTIR) spectra of the sample were obtained in transmission mode using a Bruker Vertex-80 (Bruker, Billerica, MA, USA) spectrometer. This FTIR imaging system consists of an IFS66/Sd step-scan/rapid-scan FTIR spectrometer and an infrared microscope (Hyperion 3000, Bruker, Billerica, MA, USA), as well as a matching 64 × 64 mercury cadmium telluride focal plane array (FPA) detector. FTIR spectra were collected at the mid-infrared range of 800–4000 cm^−1^ at a resolution of 8 cm^−1^ and 128 co-added scans. The spectra were collected and processed by OPUS 6.5 (Bruker, Billerica, MA, USA), and the regions within the ranges of 965–1194 cm^−1^, 1000–1200 cm^−1^, and 1600–1700 cm^−1^ were integrated. These three ranges can be used to represent CRUISER^®^, SF, wheat seed distribution, and conformational characteristics, respectively.

Mechanical tests were performed using an Instron 5966 machine (Instron, Norwood, MA, USA) at room temperature and 50% relative humidity.

The topologies of the SF–Nisin structures and the Nisin structures were recorded using a Dimension ICON atomic force microscope (AFM) FastScan system (Bruker, Billerica, MA, USA) in the ScanAsyst mode, where an aluminum reflective-coated silicon cantilever with a tip radius of 2 nm was used (k = 40 N/m).

The optical transmittance of silk fibroin film was measured using a UV-Visible-NIR spectrophotometer (Agilent Cary 5000, Agilent, Palo Alto, California, USA) with a scanning range of 380–1000 nm.

The water vapor transmittance was measured by the desiccant method.

### 2.12. Data Analysis

All data were analyzed and plotted using OriginPro 2021. The Shapiro–Wilk test was used to determine the normality of the data. For multiple comparisons, a one-way analysis of variance (ANOVA) was applied. In all cases, differences were considered significant at *p* < 0.05.

The water vapor transmittance was measured by the desiccant method.

## 3. Results

### 3.1. Analysis of Solution Stability and Film Formation in SC Composite Systems

Although lithium bromide aqueous solution is commonly used to dissolve SF, its high cost and the environmental risks associated with lithium ions prompted the use of an alternative salt-based solvent system in this study [35]. A ternary solvent system consisting of CaCl_2_, ethanol, and water was employed, with a specific mass ratio of SF to the solvent [36]. After dissolving and dialyzing through this system, an SF aqueous solution was obtained. Given that a certain amount of calcium ions is permissible in soil environments, and low concentrations of calcium ions have been shown to promote plant growth [37,38,39], we controlled the dialysis time to achieve SF solutions with varying calcium ion concentrations.

**Figure 1:**
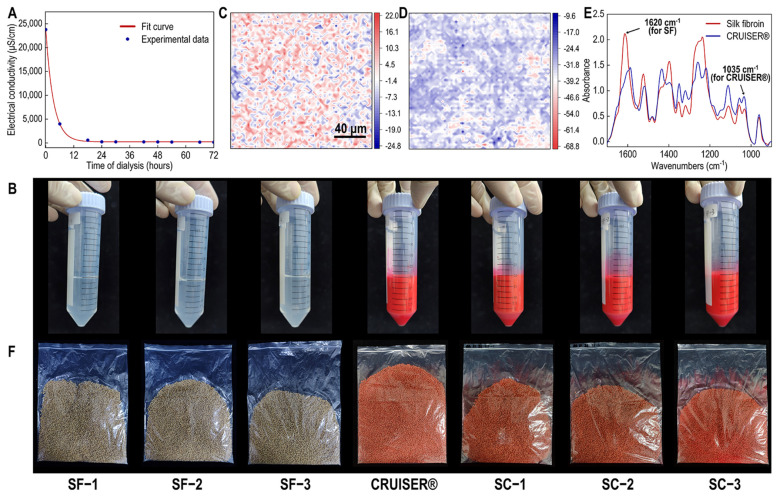
The film of silk fibroin-CRUISER^®^ (SC) composite systems. (**A**) Curve of Ca^2+^ concentration in silk fibroin (SF) solution with dialysis time. The solid red lines are fitted curves. Blue points are experimental data. (**B**) Images of different concentrations of SF solutions and SC solutions. (**C**) Fourier-transform infrared spectroscopy (FTIR) image of the SC blend. The image was integrated by the amide Ⅰ band (1600–1700 cm^−1^). (**D**) The image was integrated by the CRUISER^®^ characteristic band (965–1194 cm^−1^). (**E**) Single FTIR spectra extracted from the red (SF-rich) and blue (CRUISER^®^-rich) regions in (**C**). (**F**) Image of wheat seed coating prepared by different concentrations of SF solution and SC solution.

The effect of dialysis time on CaCl₂ concentration was assessed through the conductivity of the solution. The results indicated an exponential decay relationship between dialysis time and conductivity (Figure 1A), which could be accurately described by a fitting equation:

electrical conductivity (μS/cm) = 23,535 × e^(−t/3.27889)^ + 242.9871.

This equation allowed for the determination of the dialysis time required to achieve a specific calcium ion content. For consistency in subsequent experiments, SF solutions dialyzed for a predetermined number of hours, yielding solutions with a specific calcium ion concentration, were used as seed coating materials.

Three concentrations of SF solutions were selected to evaluate the effect of SF concentrations on film formation and coating properties. The three SF concentrations were 1%, 2%, and 5%, and were named SF-1, SF-2, and SF-3, respectively. The 1–5% concentration range has previously been found to be suitable for food preservation and coating experiments, allowing for the formation of layers with optimal thickness to ensure film stability and not hinder seed germination and growth.

For the SC composite system, three different mixing ratios are also set up to correspond with the SF system. SF solution and CRUISER^®^ were directly mixed, stirred, and labeled SC-1, SC-2, and SC-3, respectively.

As shown in Figure 1B and Figure S1, the SF solutions were uniformly mixed with CRUISER^®^ at all designated ratios, without any precipitation or flocculation observed during or after stirring. Because CRUISER^®^ is red and the SF solution is colorless and transparent, the resulting mixtures were homogeneously red with no visible phase separation, indicating good compatibility. Even after allowing the mixtures to stand for one day under natural conditions, no noticeable precipitate was observed, and the solutions remained uniform throughout the entire film formation process, with no visible precipitation of SF or other substances.

FTIR array imaging revealed the microstructure of the SC composite films. As shown in Figure 1C,D, representative bands from conventional FTIR spectra were selected for analysis: the amide I band (1600–1700 cm^−1^) for SF and the 965–1194 cm^−1^ band for CRUISER^®^. The composite coating films were subjected to micro-scale chemical imaging using a 64 × 64 FPA detector, covering an area of 260 × 260 μm with a minimum pixel size of 4 × 4 μm.

The FTIR images indicated some heterogeneity in the SC composite films. In the silk fibroin FTIR images, red and blue regions (typically 10–30 μm) were distributed throughout the matrix. Single-pixel spectra extracted from these regions revealed compositional differences: the red regions exhibited stronger amide I and II absorption but weaker absorption at 1035 cm^−1^ compared to the blue regions, suggesting that SF was enriched in the red regions while CRUISER^®^ was enriched in the blue regions (Figure 1E). Furthermore, both components were present in both the red and blue regions, indicating partial compatibility at the micro-scale of 4 × 4 μm and the absence of complete phase separation. These findings confirm that SF solutions can be effectively combined with commercial seed coating products like CRUISER^®^.

### 3.2. Evaluation of Coating Performance of SF and SC Composite Coating Systems

**Figure 2:**
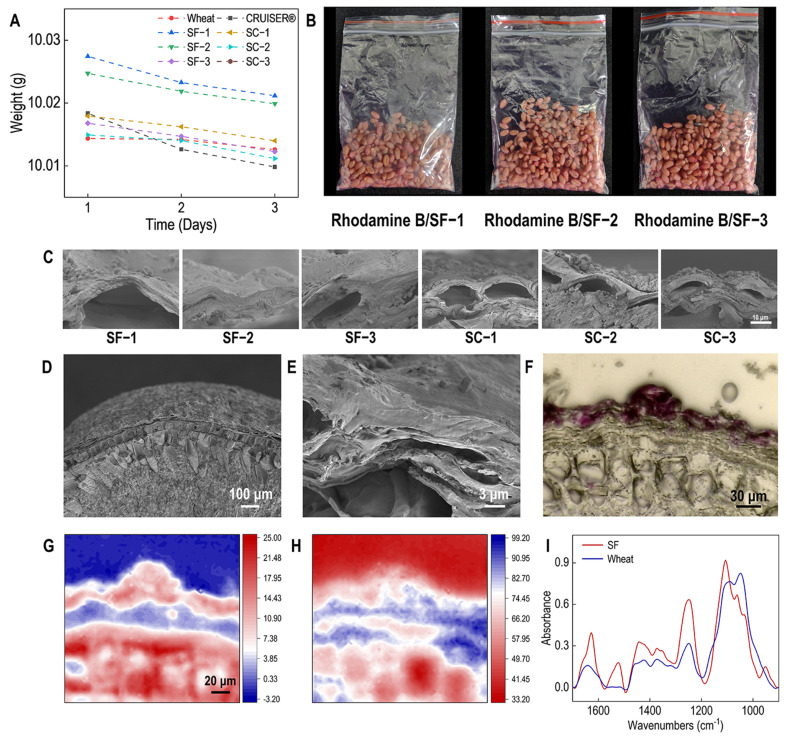
SC composite coating systems. (**A**) Changes in the weight of coated wheat seeds over time. (**B**) Images of wheat seed coating prepared by mixing SF solution of different concentrations with Rhodamine B. (**C**) Scanning electron microscope (SEM) image of cross-section of wheat seed coating prepared by SF and SC. (**D**,**E**) SEM cross-section of SC-2 coated wheat seeds. (**F**) Optical microscope image of slices of SC-2 coated wheat seeds. (**G**) FTIR image of the SC composite coating system. The image was integrated by the amide Ⅰ band (1600–1700 cm^−1^). (**H**) The image was integrated by the wheat seed characteristic band (1000–1200 cm^−1^). (**I**) Single FTIR spectra extracted from the red (SF) and blue (wheat) regions in (**G**).

In this study, wheat seeds were selected as the experimental subject, and a coating method recommended for commercial seed coatings was employed. In all experiments, the amount of coating agent was kept constant, diluted with a specific solvent, and then applied to a fixed mass of wheat seeds. After the coating treatment, the weight of the seeds was recorded daily. The results showed that three days after the coating process, the weight of the seeds stabilized, indicating that the coating film had fully formed (Figure 2A and Table S1).

As shown in Figure S2, the uncoated wheat seeds appeared brown (wheat-colored), while the pure SF coating, being colorless and transparent, made the appearance of coated seeds similar to that of uncoated seeds. This similarity made it difficult to visually assess and photograph the coating’s effectiveness. To further verify the coating performance of pure SF agents, a small amount of Rhodamine B dye (at a specific ratio) was added to the three pure SF coatings to make them visible. After dyeing, the coated seeds displayed a uniform pink color, with each seed evenly covered, indicating that all three concentrations of the SF solutions could effectively coat wheat seeds (Figure 2B). Similarly, the SC composite systems demonstrated excellent coating performance on wheat seeds. After drying, the coated wheat seeds exhibited a uniform red color with no noticeable color variation between individual seeds, and the coating thickness was nearly consistent (Figure 1F).

The coating performance of the SF and SC systems was further verified using SEM. As shown in Figure 2C, both the three SF systems and the three SC systems formed uniform coating layers on the outer surface of the wheat seeds. The coating layer in the SF systems was relatively thinner, with its thickness gradually increasing with higher SF concentrations. For example, when the concentration of SF was at a specified value, the coating thickness fell within a particular range, and the thickness increased correspondingly with higher concentrations. In contrast, the coating thickness of the SC systems remained nearly constant across the three concentration ranges, maintaining a specific thickness. This consistency was mainly due to the higher viscosity of CRUISER^®^ compared to SF, which played a determining role in the coating thickness. Additionally, observations of the interface between the wheat seeds and the coating layer revealed a tight bond with no visible gaps at the interface (Figure 2D,E).

Infrared spectroscopy imaging further confirmed the good adhesion between the coating film and the wheat seeds. As shown in Figure 2F–H, the characteristic amide absorption peaks of SF were clearly visible at the bottom of the coating layer in the SF infrared spectroscopic imaging, while the characteristic peaks of the wheat seeds were primarily detected in the lower pixel positions, indicating that the interface distance was much less than 4 μm. Additionally, the infrared spectroscopic imaging allowed for the analysis of the conformational information of SF in the coating film, as illustrated in Figure 2I. Consistent with the infrared imaging results of cast films, the SF coated on the wheat seed surface transitioned from a random coil to a β-sheet conformation upon drying. This transformation is critical for seed coating applications because SF in a random coil state is hydrophilic and relatively brittle when dry, whereas the β-sheet conformation provides a degree of hydrophobicity and enhances the material’s strength and toughness through physical cross-linking, aligning with the mechanical performance requirements for seed coatings [40,41].

### 3.3. Evaluation of the Effects of SF and SC Composite Seed Coatings on Wheat Plant and Root Growth

A planting experiment was conducted to evaluate the effects of different coating systems on wheat plant and root growth, with pure CRUISER^®^ coating (control group 1) and uncoated wheat (control group 2) serving as controls. The planting conditions included using seed trays (5.5 cm × 5.5 cm × 5 cm in length × width × height), where each tray was filled halfway with a mixture of desalinated coir brick and vermiculite (1:1 by volume), sown with 10 wheat seeds, and then covered with vermiculite. The trays were watered from the bottom, and the wheat seedlings were grown under a 12 h light/12 h dark cycle at 22 °C during the day and 18 °C at night. The results are summarized in Table S2.

**Figure 3:**
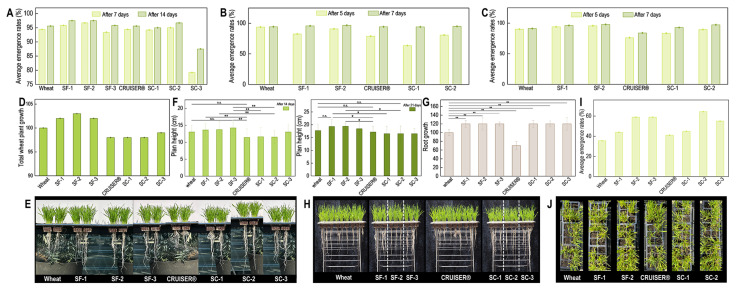
The effects of SF and SC composite seed coating on wheat plant and root growth. (**A**) The average emergence rates of seeds immediately after SF and SC coatings treatment. (**B**) The average emergence rate of seeds delayed 18 days after SF and SC coatings treatment. (**C**) The average emergence rate of seeds delayed 37 days after SF and SC coatings treatment. (**D**) The 14 days of total plant growth of wheat treated with SF and SC coatings. (**E**) Images of plant growth in (**D**). (**F**) The plant height of wheat treated with SF and SC coatings. The error bars denote standard deviations of multiple parallel experiments (n = 10). All data are mean ± SD values. The difference with *p* < 0.05 was considered statistically significant (* *p* < 0.05, ** *p* < 0.01, n.s.: not significant). (**G**) The 21 days of root growth of wheat treated with SF and SC coatings. The error bars denote standard deviations of multiple parallel experiments (n = 10). All data are mean ± SD values. The difference with *p* < 0.05 was considered statistically significant (* *p* < 0.05, ** *p* < 0.01, n.s.: not significant). (**H**) Images of root growth in (**G**). The white dashed lines are used to divide SF-1, SF-2, SF3, and SC-1, SC-2, and SC-3. (**I**) The average emergence rate of seeds under cold stress conditions after SF and SC coating treatment. (**J**) Images of seed emergence in (**I**).

Comparing the average emergence rates of seeds treated with SF and SC coatings with those of the control groups revealed no significant differences (Figure 3A). For example, average emergence rates (93.4–97.5%) 7 days post-sowing was close to that of the uncoated group (94.4%), and the average emergence rates 14 days post-sowing were mostly over 95%, also close to that of the uncoated group (95.6%). Among them, the emergence rate of SC-3 was abnormal, and its effect would be comprehensively evaluated in combination with other aspects.

As shown in Figure 3D–H, the SF-containing seed coatings enhanced wheat plant and root growth. To facilitate quantitative comparison, we not only measured plant height directly but also scored plant growth and root growth through visual assessment. The results showed that wheat seedlings treated with pure SF coatings exhibited superior plant height and root growth on days 14 and 21 post-sowing. On day 14, the average plant height for SF-1 and SF-2 reached 14 cm, higher than the 13 cm observed in the uncoated group. By day 21, the average plant height for SF-1 and SF-2 further increased to 19 cm.

Regarding root growth, the SF-1 to SF-3 treatments were significantly better than that of the uncoated group, indicating that SF coatings promote root growth (*p* < 0.01). This enhancement may be related to the adsorption capacity of SF for water and nutrients, facilitating nutrient uptake by the roots. Similarly, SC coatings showed advantages in plant height and root growth compared to pure CRUISER^®^ and uncoated groups, especially at day 21, and the root growth of SC treatments was significantly better than that of the CRUISER^®^ group and the uncoated group (*p* < 0.01).

In the seed emergence experiment under cold stress conditions, seeds treated with the SF and SC coatings exhibited higher average emergence rates, reaching up to 64.4% (SC-2), which was much better than the CRUISER^®^-coated (41.1%) and uncoated groups (35.6%) (Figure 3I,J). This indicates that the protective layer formed by SF mitigated the impact of low temperatures on seed viability, thus enhancing cold resistance.

Under delayed sowing conditions, the SF-containing seed coatings also demonstrated excellent performance (Figure 3B,C). For example, seeds that were sown on day 18 post-coating showed slightly lower average emergence rates than the uncoated group five days after sowing but exceeded 95% seven days post-sowing. Seeds that were sown on day 37 achieved an average emergence rate of over 92.8%, even surpassing the uncoated group’s 91.1%.

Notably, in the sowing experiment, SF-3 had a significantly lower average emergence rate than SF-1 and SF-2, potentially due to the thicker coating layer resulting from the higher SF concentration, which may have hindered gas and water exchange. Nonetheless, SF-3 still showed a relatively high emergence rate (58.9%) under cold stress conditions, suggesting that the protective effect of SF is more pronounced under adverse conditions. Future research could further optimize the SF concentration to balance coating thickness and breathability, thereby improving emergence outcomes.

### 3.4. Optimization of the SF Composite Seed Coating Design

The evaluation of wheat plant and root growth demonstrated that seeds coated with pure SF exhibited significant advantages in promoting plant and root growth, with certain indicators (such as plant growth and plant height) even surpassing those of the SC composite systems (Figure 3D,F). However, SF lacks inherent antibacterial properties, meaning that seeds coated solely with SF require higher preservation standards during storage compared to those coated with CRUISER^®^ or SC composites. For instance, seeds coated with pure SF need to be stored in a sealed environment, as they may develop mold within approximately two months under ordinary natural storage conditions. In contrast, seeds coated with CRUISER^®^ or SC composites can be stored for over a year in a naturally dry environment, which meets the shelf-life requirements for many fast-selling seeds.

However, fungicides commonly used in CRUISER^®^ and other seed coating agents, such as thiamethoxam, fludioxonil, and tebuconazole, exhibit high toxicity, presenting a major environmental issue for current commercial seed coatings [42,43,44,45,46]. To meet the demands of sustainable agriculture, there is an urgent need to find alternatives to these fungicides. A potential solution explored in this study is the combination of SF with antimicrobial peptides to enhance the antibacterial performance of SF-based seed coatings.

In this research, Nisin was selected as the antimicrobial peptide. Nisin is a polypeptide substance produced by *Lactococcus lactis*, consisting of 34 amino acid residues with a molecular weight of approximately 3500 Da [30]. It exhibits inhibitory activity against most Gram-positive bacteria, particularly against the spores of *Bacillus* species, and is widely used as a preservative in the food industry [47,48]. Nisin is safe, non-toxic, does not disrupt the normal human microbiota, and does not induce resistance, making it an ideal natural preservative [49,50]. Additionally, Nisin can be produced on a large scale at a low cost through *Lactococcus lactis* fermentation, providing a significant advantage over other antimicrobial peptides that require synthetic biology techniques for production [51].

**Figure 4:**
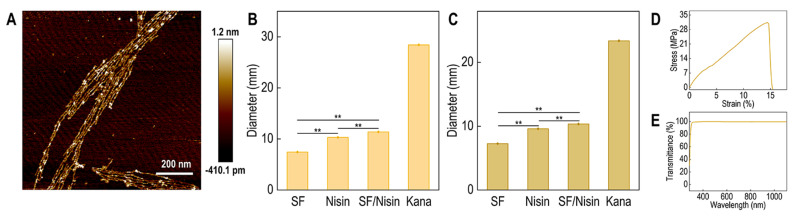
SF–Nisin composite coating systems. (**A**) The atomic force microscope (AFM) image of SF–Nisin. (**B**) The diameter of inhibition zone of the SF–Nisin composite system on Staphylococcus aureus. The error bars denote standard deviations of multiple parallel experiments (n = 5). All data are mean ± SD values. The difference with *p* < 0.05 was considered statistically significant (** *p* < 0.01, n.s.: not significant). (**C**) The diameter of inhibition zone of the SF–Nisin composite system on Bacillus subtilis. The error bars denote standard deviations of multiple parallel experiments (n = 5). All data are mean ± SD values. The difference with *p* < 0.05 was considered statistically significant (** *p* < 0.01, n.s.: not significant). (**D**) Stress–strain curves of SF film. (**E**) The transmittance of SF film.

As shown in Figure 4A and Figure S3, Nisin can form a uniform mixture with the SF solution, and AFM observations revealed that Nisin is evenly dispersed within the molecular network of SF on a molecular scale. Compared to the pure SF solution, the SF–Nisin composite solution exhibited significantly enhanced antibacterial activity against *Staphylococcus aureus* and *Bacillus subtilis* (*p* < 0.01). As illustrated in Figure 4B,C, the inhibitory effect of the SF–Nisin composite system on *Staphylococcus aureus* increased by 1.53-fold, while its inhibitory effect on *Bacillus subtilis* increased by 1.42-fold. Furthermore, SF film has excellent physical and chemical properties, such as good film-forming ability, mechanical strength, and barrier performance (Figure 4D,E), and a water vapor transmittance of 108.43 g/(m^2^ day).

The results of the inhibition zone experiment (Figure 4B,C) showed that the inhibition zones of the SF–Nisin composite were larger for the two tested bacterial strains compared to those of the pure SF coating. This enhancement is due to Nisin’s ability to penetrate bacterial cell membranes, creating pores that lead to the leakage of cytoplasmic contents and subsequent cell lysis and death [52]. Additionally, the experiments indicated that the antibacterial effect of the composite film was superior to that of Nisin alone, potentially because the macromolecular network of SF provided a carrier for the slow release of Nisin, while pure Nisin tends to burst-release and dissipate quickly in aqueous environments, making it challenging to achieve sustained antibacterial action.

In summary, our study demonstrates that the simple combination of SF and Nisin significantly improves the antibacterial properties of SF-based seed coatings, addressing the shortcomings in its antibacterial performance (*p* < 0.01). Future research could develop more complex and diverse composite systems based on this approach to gradually replace the toxic components in current commercial seed coatings, ultimately achieving a green and environmentally friendly design for seed coating agents.

## 4. Discussion

This study demonstrates the potential of SF as an effective and sustainable seed coating agent, offering a promising alternative to conventional chemical-based coatings. The systematic evaluation of SF and its composite systems with CRUISER^®^ and Nisin highlights several key advantages in seed protection, germination, and growth enhancement.

The results show that SC composites exhibit excellent solution stability and film-forming capabilities, largely due to the formation of stable β-sheet structures, which significantly improve the coating’s mechanical properties and durability. This synergy enhances the uniformity and strength of the coating, providing robust physical protection to seeds.

The findings also reveal that SF-based coatings improve wheat seedling emergence rates and plant height, especially under cold stress, suggesting enhanced environmental adaptability. At the same time, SF-based coatings significantly increased root growth (*p* < 0.01). It may be that SF is conducive to the root’s absorption of water and nutrients. However, this study identifies limitations in the antimicrobial efficacy of pure SF, which was addressed by incorporating Nisin to significantly improve antibacterial properties (*p* < 0.01). The SF–Nisin composite demonstrated sustained inhibition of bacterial pathogens, providing an eco-friendly solution to the limitations of chemical fungicides.

Future work should focus on optimizing the SF concentration and exploring more complex combinations with other natural materials, such as chitosan or plant essential oils, to further enhance the coating’s multifunctional properties. Additionally, large-scale field trials are needed to validate the practical effectiveness and economic viability of these sustainable seed coatings in diverse agricultural settings. Overall, this research provides a foundation for the development of green, eco-friendly seed coatings, paving the way for safer and more sustainable agricultural practices.

## Figures and Tables

**Table 1:** Different proportions of seed coating agents.

	CRUISER^®^ (mL)	SF (mL)	H_2_O (mL)
CRUISER^®^	2	/	18
SF-1	/	1	19
SF-2	/	2	18
SF-3	/	5	15
SC-1	2	1	17
SC-2	2	2	16
SC-3	2	5	13

The “/” indicates that the component is not added.

## Data Availability

The original contributions presented in this study are included in the article/Supplementary Materials; further inquiries can be directed to the corresponding authors.

## Supplementary Materials

The following supporting information can be downloaded at https://www.mdpi.com/article/10.3390/polym16233281/s1, Figure S1. Film formation of silk fibroin (SF) solution and silk fibroin-CRUISER^®^ (SC) solution. Figure S2. Wheat seeds and wheat seeds treated with SF coatings. Figure S3. The atomic force microscope image of Nisin solution. Table S1. The weight (g) of the wheat seeds for different days after coating. Table S2. The effects of SF and SC composite seed coatings on wheat plant and root growth.
