# Supplementary material for: Silk Fibroin Seed Coatings: Towards Sustainable Seed Protection and Enhanced Growth
Source: Polymers (Basel). 2024 Nov 25;16(23):3281. doi: 10.3390/polym16233281 (PMC11644567; doi:10.3390/polym16233281)
Supplement: Supplementary file 1 [file polymers-16-03281-s001.zip › polymers-3289307-supplementary.pdf]

Supporting Information

Silk Fibroin Seed Coatings: Towards Sustainable Seed Protec-tion and Enhanced Growth

Feng Jin†, Zhengrong Guan†, Jiahao Zhang, Zhigang Qu, Shengjie Ling, Leitao Cao, Jing Ren, Ruoxuan Peng**

Supporting information contains:

Figure S1-S3

Table S1-S2

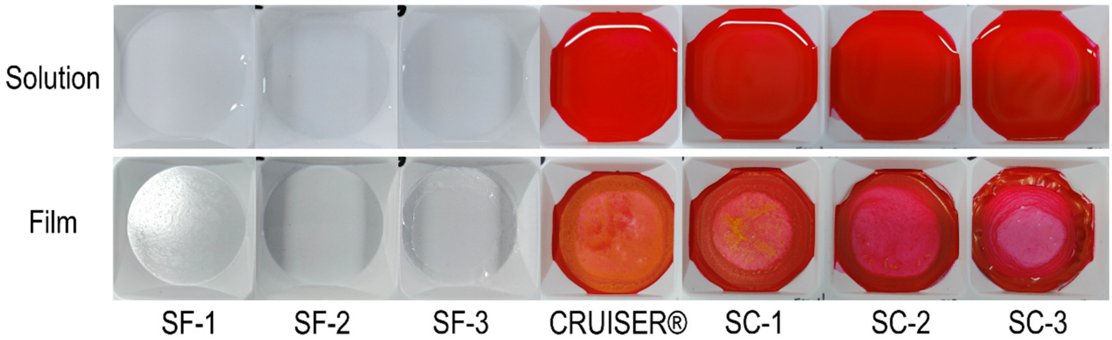

Figure S1. Film formation of silk fibroin (SF) solution and silk fibroin-CRUISER® (SC) solution.

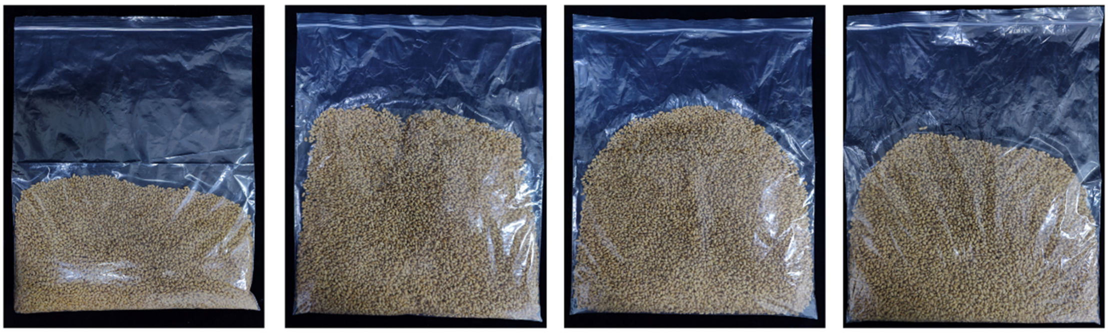

Figure S2. Wheat seeds and wheat seeds treated with SF coatings.

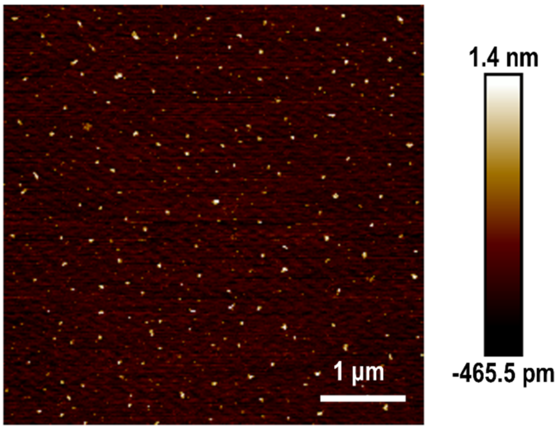

Figure S3. The atomic force microscope image of Nisin solution.

Table S1. The weight (g) of the wheat seeds for different days after coating.

		Wheat	SF-1	SF-2	SF-3	CRUISER®	SC-1	SC-2	SC-3
**1th day**	1	10.0233	10.0471	10.0390	10.0111	10.0275	10.0138	10.0158	10.0365
	2	10.0198	10.0037	10.0194	10.0347	10.0117	10.0246	10.0170	10.0284
	3	10.0000	10.0315	10.0158	10.0046	10.0159	10.0154	10.0120	10.0055
**2nd day**	1	10.0243	10.0426	10.0363	10.0090	10.0189	10.0111	10.0156	10.0351
	2	10.0192	10.0002	10.0170	10.0325	10.0076	10.0236	10.0161	10.0277
	3	9.9991	10.0270	10.0123	10.0026	10.0114	10.0140	10.0104	10.0042
**3rd day**	1	10.0218	10.0416	10.0354	10.0067	10.0153	10.0084	10.0133	10.0322
	2	10.0178	9.9966	10.0138	10.0294	10.0052	10.0212	10.0132	10.0236
	3	9.9982	10.0253	10.0105	10.0007	10.0090	10.0124	10.0070	10.0018

Table S2. The effects of SF and SC composite seed coatings on wheat plant and root growth.

		Wheat	SF-1	SF-2	SF-3	CRUISER®	SC-1	SC-2	SC-3
**Emergence rates (%)**	After 7 days	94.4	95.8	96.7	93.4	94.4	94.2	95	79.2
	After 14 days	95.6	97.5	97.5	95.8	95.6	95	96.7	87.5
**Plant growth**	After 14 days	100	102	103	102	98	98	98	99
	After 21 days	100	101	101	/	99	/	/	/
**Plant height (cm)**	After 14 days	13	14	14	14	12	12	12	13
	After 21 days	18	19	19	18	17	16.5	16.5	16.5
**Root growth**	After 21 days	100	120	120	120	70	120	120	120
**Emergence rates under cold stress (%)**		35.6	43.9	58.9	58.9	41.1	45	64.4	55
**Emergence rates on day 18 post-coating (%)**	After 5 days	93.9	82.5	90.8	/	78.9	63.6	80.8	/
	After 7 days	94.4	95.8	96.7	/	94.4	94.2	95	/
**Emergence rates on day 37 post-coating (%)**	After 5 days	90	93.9	95.6	/	76.1	83.3	89.4	/
	After 7 days	91.1	96.1	97.8	/	83.9	92.8	97.2	/
